# Just call it “treatment”

**DOI:** 10.1186/1940-0640-7-10

**Published:** 2012-06-09

**Authors:** Peter D Friedmann, Robert P Schwartz

**Affiliations:** 1 Warren Alpert Medical School, Brown University, Providence, RI; Providence Veterans Affairs Medical Center, Providence, RI; and the Department of Medicine, Rhode Island Hospital, 593 Eddy Street, Providence, RI, 02903, USA; 2 Friends Research Institute, 1040 Park Avenue, Suite 103, Baltimore, MD, 21201, USA

**Keywords:** Buprenorphine, Behavioral counseling, Methadone, Opioid dependence, Substance abuse counseling, Substance abuse treatment.

## Abstract

Although many in the addiction treatment field use the term “medication-assisted treatment” to describe a combination of pharmacotherapy and counseling to address substance dependence, research has demonstrated that opioid agonist treatment alone is effective in patients with opioid dependence, regardless of whether they receive counseling. The time has come to call pharmacotherapy for such patients just “treatment”. An explicit acknowledgment that medication is an essential first-line component in the successful management of opioid dependence.

## 

The recently published National Institute on Drug Abuse Clinical Trials Network’s Prescription Opiate Treatment Study (POATS) [1] found that only 6.6% of prescription-opioid dependent participants had minimal or no opioid use following brief treatment with buprenorphine/naloxone (BUP/NX). Patients enrolled in that trial who returned to opioid use on discontinuation of BUP/NX resumed BUP/NX for an extended period. Although 49.2 % of those patients who resumed BUP/NX had a successful outcome at the final week of the extended BUP/NX treatment, the success rate dropped to 8.6% at eight weeks after a two-week dose taper. In neither case did individual opioid dependence counseling (45–60 minute weekly sessions with a trained mental health or substance abuse professional) provide additional benefit over standard medical management (15–20 minute visits with a physician certified to prescribe BUP/NX).

Increasingly, practitioners, administrators, and policymakers in the addiction treatment field have taken to using the terms “medication-assisted treatment” or “medication-assisted recovery” to describe the combination of pharmacotherapy with counseling and/or recovery work. Recovery-movement traditionalists have maintained that addiction remission is not genuine if produced through use of medication alone, because the person has not undergone the interpersonal and spiritual changes deemed necessary for lasting recovery. The terms medication-assisted treatment and medication-assisted recovery manifest this perspective. Such terms bespeak an implicit judgment that medication is only an adjunct to the “truly effective components” of counseling and recovery work.

Terminology is meaningful in a field because it both reflects and influences the beliefs of practitioners. The view that pharmacotherapy-induced remission is less valuable than “real” recovery stigmatizes patients, providers, and the therapy itself. The view of medication as a temporary adjunct opens the door for rejection of patients on medication at some self-help meetings, time limits on insurance coverage for addiction medication, and preference for medication tapering on the part of patients, practitioners and criminal justice professionals, despite evidence that this approach leads to inferior and sometimes adverse outcomes, including death [2]. Such views are contrary to the modern perspective on opioid dependence, that many patients should be treated as having a chronic neurobehavioral brain disorder.

Although one earlier clinical trial conducted among veterans suggested that adding counseling to methadone increased opioid agonist treatment (OAT) efficacy [3], much research prior to the POATS has demonstrated that pharmacotherapy alone is effective treatment for opioid dependence with minimal to no drug-abuse counseling. A recent Cochrane systematic review of the literature found that OAT without counseling is more effective than being waitlisted for treatment or receiving psychosocial treatment with or without placebo [4]. In addition, randomized clinical trials have provided strong evidence for the effectiveness of directly administered methadone without drug abuse counseling for one month [5], four months [6], and six months [7].

Throughout the world, OAT is commonly delivered with minimal or no counseling beyond standard medication management, with rates of treatment retention and improvement in illicit drug use comparable to OAT with counseling [8-12]. In the United States, a study on office-based buprenorphine treatment also found that intensive counseling with OAT was no more effective than opioid agonist pharmacotherapy with standard medication management [13].

The POATS findings and other rigorous studies demonstrate that OAT is effective in suppressing opioid use as long as it is maintained, and that a tapering detoxification strategy, regardless of duration, fails the great majority of opioid-dependent patients [14-16]. As with the treatment of hypertension or diabetes, as long as the patient takes the medication, the disorder’s manifestations are mitigated; when the medication is stopped, those manifestations recur [17]. For many patients seeking treatment for opioid dependence, drug abuse counseling does not appear to add any measurable improvement in outcome beyond prescribed buprenorphine with standard medication management delivered in an office-based setting [1], or direct administration of methadone without counseling in an opiate treatment program [7,18].

It should not be construed that drug abuse counseling is without value. Such counseling should be offered to patients, but patient resistance to counseling should not be a barrier to receiving highly effective medication, such as methadone or buprenorphine, any more than insulin should be withheld from diabetic patients who refuse dietary counseling. Perhaps for this reason, the World Health Organization has called effective treatment for opioid dependence *psychosocially-assisted pharmacotherapy*[19].

*Counseling-assisted pharmacotherapy* has also been suggested as a term that reflects the true relative effectiveness of these treatment modalities [20]. However, other medical disciplines do not use the modifer “-assisted” to describe multimodal treatment. Type-2 diabetics take medication and get counseling about weight loss, diet and exercise; all are important, and none is viewed as “assisting.” The time has come to call medication therapy for addiction just “treatment”—an explicit acknowledgment that pharmacotherapy is an essential component and common first-line treatment for opioid dependence.

## Competing interests

Dr. Friedmann declares that Alkermes has donated medication for a NIDA/National Institutes of Health (NIH) funded study for which he is principal investigator. Dr. Schwartz declares that Reckitt-Benckiser has donated medication for a NIDA/NIH funded study for which he is a co-investigator.
